# Basic fibroblast growth factor reduces scar by inhibiting the differentiation of epidermal stem cells to myofibroblasts via the Notch1/Jagged1 pathway

**DOI:** 10.1186/s13287-017-0549-7

**Published:** 2017-05-16

**Authors:** Peng Wang, Bin Shu, Yingbin Xu, Jiayuan Zhu, Jian Liu, Ziheng Zhou, Lei Chen, Jingling Zhao, Xusheng Liu, Shaohai Qi, Kun Xiong, Julin Xie

**Affiliations:** 1 Department of Burn Surgery, First Affiliated Hospital of Sun Yat-Sen University, No. 58, 2nd Zhongshan Road, Yuexiu District, Guangzhou, Guangdong Province 510080 People’s Republic of China; 2 Department of Anatomy and Neurobiology, School of Basic Medical Sciences, Central South University, Changsha, Hunan Province 410013 People’s Republic of China

**Keywords:** Scar, Basic fibroblast growth factor, Epidermal stem cells, Myofibroblasts, Notch1/Jagged1 pathway

## Abstract

**Background:**

Basic fibroblast growth factor (bFGF) plays an important role in promoting wound healing and reducing scar, but the possible molecular mechanisms are still unclear. Our previous studies have found that activating the Notch1/Jagged1 pathway can inhibit the differentiation of epidermal stem cells (ESCs) to myofibroblasts (MFB). Herein, we document that bFGF reduces scar by inhibiting the differentiation of ESCs to MFB via activating the Notch1/Jagged1 pathway.

**Methods:**

In in-vitro study, ESCs were isolated from 10 neonatal SD rats (1–3 days old), cultured in keratinocyte serum-free medium, and divided into six groups: bFGF group, bFGF + SU5402 group, bFGF + DAPT group, siJagged1 group, bFGF + siJagged1 group, and control group. Jagged1 of the ESCs in the siJagged1 group and bFGF + siJagged1 group was knocked down by small-interfering RNA transfection. Expression of ESC markers (CK15/CK10), MFB markers (α-SMA, Collagen I, Collagen III), and Notch1/Jagged1 components (Jagged1, Notch1, Hes1) was detected by FCM, qRT-PCR, and western blot analysis to study the relationships of bFGF, ESCs, and Notch1/Jagged1 pathway. In in-vivo study, the wound healing time and scar hyperplasia were observed on rabbit ear scar models. The quality of wound healing was estimated by hematoxylin and eosin staining and Masson staining. Expression of ESC markers, MFB markers and Notch1/Jagged1 components was elucidated by immunohistochemistry, immunofluorescence, and western blot analysis.

**Results:**

The in-vitro study showed that bFGF could significantly upregulate the expression of ESC markers and Notch1/Jagged1 components, while downregulating the expression of MFB markers at the same time. However, these effects could be obviously decreased when we knocked down Jagged1 or added DAPT. Similarly, in in-vivo study, bFGF also exhibited its functions in inhibiting the differentiation of rabbit ESCs to MFB by activating the Notch1/Jagged1 pathway, which improved the wound healing quality and alleviated scar significantly.

**Conclusion:**

These results provide evidence that bFGF can reduce scar by inhibiting the differentiation of ESCs to MFB via the Notch1/Jagged1 pathway, and present a new promising potential direction for the treatment of scar.

## Background

Scar is one of the most common complications of wound healing, which not only traps patients into disfigurement, functional impairment, and physical and mental agony, but also makes them a heavy burden for their families and society [1]. Although current studies have discovered some treatments, such as pressure therapy, topical silicone gel, and so forth, the curative effects are still unsatisfactory [2]. Therefore, further research on the mechanism of scar is extremely crucial.

It has been widely confirmed that the main causes of scar are the lack of epidermal stem cells (ESCs) and the excessive hyperplasia of myofibroblasts (MFB) [3, 4]. Located in the basal layer of the epidermis and the follicle bugle of hair, ESCs possess a strong proliferation and differentiation potential [5]. In physiological conditions, ESCs keep the normal structure and function of skin and repair damage by proliferation, migration, and differentiation [6]. Theoretically, all skin lesions can be repaired by ESCs. However, in the case of severe skin appendage damage, such as follicle and sweat gland injury, the scar tissue, which lacks hair and sweat glands, is widespread [7]. We also found that the number of ESCs in scar was significantly lower than in normal skin, while MFB were significantly higher [8]. This phenomenon implied that the proliferation decrease and abnormal differentiation of ESCs can be an important reason for scar.

The proliferation and differentiation of ESCs mainly depend on the regulation of stem cell niches, which include growth factor, extracellular matrix and signaling pathways, and so forth [9]. Among these, the Notch pathway mediated by Jagged1 plays an important role [10]. Our previous research found that activating the Notch1/Jagged1 pathway can promote ESC proliferation and inhibit the cells’ differentiation to MFB [11, 12]. The specific mechanism is as follows. With Jagged1 binding to Notch1 receptors, the Notch intracellular domain (NICD) can be released and translocated to the nucleus and can induce the activating of target genes *Hairy* and enhancer of split-1 (*Hes1*), which play a key role in promoting proliferation and inhabiting differentiation of stem cells [13].

Years of clinical observation have documented that basic fibroblast growth factor (bFGF) can promote wound healing and reduce scarring [14], but how it regulates the proliferation and differentiation of ESCs and the possible molecular mechanism remains unclear. In our previous study, we found that bFGF could promote ESC proliferation and inhibit the cells’ differentiation to MFB [15]. These effects are consistent with the Notch1/Jagged1 pathway. Therefore, we implemented the study to determine whether bFGF could regulate the proliferation and differentiation of ESCs by activating the Notch1/Jagged1 pathway and ultimately promote wound healing and reduce scarring. The results of our study not only indicate a relation of bFGF, the Notch1/Jagged1 pathway, and ESCs in wound healing, but also reveal the formation of scar in a new view, which may help us to explore new approaches to prevent and treat scar.

## Methods

### Animal sources and ethics statement

All animal experiments were approved by the Institutional Animal Care and Use Committee at Sun Yat-Sen University and were performed according to National Institutes of Health guidelines. Sprague–Dawley (SD) rats (fetal, age: 1–3 days, weight: 8–10 g, grade: SPF) and New Zealand rabbits (female, age: 8–10 weeks, weight: 2–2.5 kg, grade: clean) were obtained from the Experimental Animal Center of Sun Yat-Sen University (license: SYXK 2016-0112) and kept in standard conditions according to the regulation of ethics committee of the Medical Sciences Department.

### Isolation and culture of ESCs

ESCs were isolated from the back of newborn SD rats’ skin. Briefly, the skin samples were taken from the back of fetal SD rats and cut into pieces (approximately 0.3 × 0.3 cm^2^). After incubation in 0.5% Dispase II (17105041; Gibco) in PBS at 4 °C overnight, the epidermal sheets were carefully separated from the dermis and digested in 0.25% trypsin (25200-056; Gibco) at 37 °C for 20 minutes. The trypsin was inactivated in Dulbecco’s modified Eagle’s medium (DMEM, 12100-046; Gibco) containing 10% FBS. Followed by filtering and centrifuge, the cells were resuspended in keratinocyte serum-free medium (K-SFM, 17005042; Gibco) and seeded at a density of 10^5^ cells/cm^2^ in flasks coated with 100 μg/ml collagen IV (ab6586; Abcam) to adhere for 15 minutes at 37 °C. The rapidly adhering cells were collected and cultured in K-SFM medium at 37 °C in 5% CO_2_. When the culture reached 70–80% confluence, the cells were digested and passaged at a ratio of 1:2 [16]. Meanwhile, the cells were identified to be ESCs with integrin-α6^bri^ (3750S; CST, BOS, USA) and CD71^dim^ (553264; BD) by immunofluorescence staining [17].

### Cell treatment

ESCs of SD rats were redivided into groups of bFGF (10 ng/ml, PHG0264; Gibco), bFGF + SU5402 (10 μmol/L, PK-CA577-1645-05; PromoCell, Germany), bFGF + DAPT (20 μmol/L, ab120633; Abcam, UK), siJagged1, bFGF + siJagged1, and control. The control group was treated with K-SFM medium only. The siJagged1 group was transfected by specific small-interfering RNA (siRNA) for Jagged1, to knock down Jagged1 ligand [18]. SU5402 and DAPT are the specific inhibitors of bFGF receptor and Notch signaling respectively [19, 20].

### Transient transfection with siRNA and Jagged1 knockdown

The sequences of siRNA for Jagged1 (5′-CAGCGAAUUGAGGAAUCUGTT-3′) and the negative control (5′-UUCUCCGAACGUGUCACGUTT-3′) were designed and synthesized by RiboBio (Guangzhou, China). Before transfection, the cells (3 × 10^5^ cells/well) were plated in a six-well plate (Nest Biotech, Shanghai, China) and cultured in fresh K-SFM medium for 24 hours. At approximately 50% cell confluence, the designed siRNA was transfected to cells by TurboFect siRNA Transfection Reagent (Fermentas, Vilnius, Lithuania) according to the manufacturer’s protocol. The knock-down efficiency of Jagged1 was detected by qRT-PCR and western blot (Fig. 2a, b). The transfected cells were harvested at 72 hours post transfection for protein extraction and further study.

### Flow cytometry analysis

ESCs (1 × 10^6^/ml) were trypsinized and suspended in 2% BSA/PBS (16000-044; Gibco) after 10 days of culture. After centrifuge and resuspension, the cells were incubated for 2 hours at room temperature with the following primary antibodies: anti-CK10 (1:100, ab9026; Abcam), anti-CK15 (1:100, ab52816; Abcam), and anti-α-SMA (1:20, ab32575; Abcam). Followed by centrifuge and washing, the resuspended cells were added in FITC-labeled secondary antibody IgG (1:500, ab6785; Abcam), and incubated for 30 minutes. The expression of CK10, CK15, and α-SMA was detected by BD Accuri C6 (BD, USA). Independent experiments were done in triplicate.

### Quantitative real-time PCR

Total RNA was extracted from the rats’ ESCs by Trizol Reagent (Invitrogen, CA, USA), and transcribed into cDNA using the PrimeScript RT reagent Kit (TaKaRa, Dalian, China). Quantitative real-time PCR (qRT-PCR) was performed with SYBR Premix ExTaq (TaKaRa, Dalian, China) and the primer sequences presented in Table 1. The qRT-PCR reactions were processed in the Stratagene Mx3000P real-time PCR system (Agilent Technologies, CA, USA). GAPDH (ab9485; Abcam) was used as internal control for mRNA quantification. The relative expression ratio of mRNA was calculated by the 2^−ΔΔCT^ method. PCR reactions for each gene were repeated three times.

**Table 1:** Primer sequences for quantitative real-time PCR

Gene name	Forward	Reverse
*α-SMA*	5′-CATCACCAACTGGGACGACA-3′	5′-TCCGTTAGCAAGGTCGGATG-3′
*Collagen I*	5′-GTACATCAGCCCAAACCCCA-3′	5′-CAGGATCGGAACCTTCGCTT-3′
*Collagen III*	5′-ATATGTGTCTGCGACTCGGG-3′	5′-GGGCAGTCTAGTGGCTCATC-3′
*Jagged1*	5′-TGAGGACTACGAGGGCAAGA-3′	5′-GCACCCCTTCAGGAGTATCG-3′
*Notch1*	5′-CAATGGCACAGGGGCTATGA-3′	5′-TTAGCGGGTTGTACTGGCTG-3′
*Hes1*	5′-AGCGCTACCGATCACAAAGT-3′	5′-ACGTCCCCTTTACTTGGCTT-3′
*GAPDH*	5′-GGGGCTCTCTGCTCCTCCCTG-3′	5′-CGGCCAAATCCGTTCACACCG-3′

### Protein extraction and western blot analysis

Total proteins of rats’ ESCs or rabbits’ tissues were extracted with the ProteoPrep® Total Protein Extraction Kit (PROTTOT-1KT; Sigma), and the protein concentration was determined by the BCA Assay Kit (23225; Pierce). After boiling for 10 minutes, equal amounts of protein extract (50 μg) were subjected to electrophoresis in 10% SDS-PAGE gels at 100 V for 2 hours, and then transferred to PVDF membranes (Millipore) at 100 V for 90 minutes. The membranes were blocked with 5% nonfat milk in TBST (0.1 M, pH 7.4) and incubated overnight at 4 °C with one of the following primary rabbit anti-mouse antibodies: anti-α-SMA (1:1000, ab32575; Abcam), anti-Collagen I (1:1000, ab34170; Abcam), anti-Collagen III (1:1000, ab7778; Abcam), anti-Jagged1 (1:500, ab7771; Abcam), anti-Notch1 (1:1000, ab52627; Abcam), and anti-Hes1 (1:1000, ab71559; Abcam). After washing with TBS/Tween-20 solution, the membranes were incubated with peroxidase-conjugated secondary antibody IgG (1:2000, ab6721; Abcam). Finally, protein bands were detected by the Odyssey infrared imaging system (LI-COR Biosciences) and analyzed by Image Pro-Plus 6.0 software (Media Cybernetics). Quantitative western blot measurements of target protein were normalized by corresponding measures of GAPDH derived from the same samples in each blot. Independent experiments were done in triplicate.

### Animal study

To study the function of ESCs in scar, the rabbit ear scar model was adopted [21]. Before injury, 10 New Zealand rabbits (8–10 weeks old) were injected intraperitoneally with 50 mg/kg 5-bromodeoxyuridine (BrdU, B-9285; Sigma) four times every 12 hours to identify ESCs. Because ESCs possess a longer period for division, they should be the only cells in skin to retain the BrdU label after a 60-day chase period [22]. Two full-thickness wounds with a diameter of 2 cm were then made in the skin of each ear and the rabbits were divided into four groups randomly. bFGF (10 ng/ml), bFGF (10 ng/ml) + DAPT (20 μmol/L), and TGF-β1 (10 ng/ml) were applied respectively to the wound daily until wound closure, while the control group was only cleaned by saline. At the same time, we recorded the wound healing time, photographed the wound, measured the wound areas and the thickness of scar tissue using National Institutes of Health ImageJ software, and calculated the residual wound area rate and scar index regularly. The formula for calculating the residual wound area rate is [22]:

$$ \mathrm{Residual}\ \mathrm{wound}\ \mathrm{area}\ \mathrm{rate} = \left[\left(\mathrm{day}\  n\ \mathrm{area}\right)\ /\ \left(\mathrm{day}\ 0\ \mathrm{area}\right)\right] \times 100\%\kern2em \left( n = 0,\ 7,\ 14,\ 21,\ \mathrm{or}\ 30\right). $$

The formula for calculating the scar index is:

$$ \mathrm{Scar}\ \mathrm{index} = \left[\left(\mathrm{thickness}\ \mathrm{of}\ \mathrm{the}\ \mathrm{scar}\ \hbox{--}\ \mathrm{thickness}\ \mathrm{of}\ \mathrm{the}\ \mathrm{adjacent}\ \mathrm{normal}\ \mathrm{skin}\right)\ /\ \left(\mathrm{thickness}\ \mathrm{of}\ \mathrm{adjacent}\ \mathrm{normal}\ \mathrm{skin}\right)\right] \times 100\%. $$

The wound tissues at 0, 7, 14, 30, and 60 days were harvested and separated into two halves across the center: one half was processed for histological analysis and immunofluorescence analysis, and the other was rapidly frozen in liquid nitrogen for protein analysis.

### Histological analysis and immunohistochemistry staining

For further histological study, skin tissue samples were routinely fixed with formalin, embedded in paraffin, and sectioned at 4 μm thick. The sections of each group at 7, 14, 30, and 60 days were deparaffinized and stained with hematoxylin and eosin (H&E) and Masson, and were examined under blindfold conditions with standard light microscopy (OLYMPUS, Japan) to observe the skin epidermis, dermis, accessories, inflammation, and scar tissue.

The paraffin-embedded fixed tissue sections of each group were deparaffinized and rehydrated. Following antigen retrieval, the sections were blocked with 2% goat serum in PBS for 20 minutes and then incubated with mouse monoclonal anti-α-SMA (1:50, ab7817; Abcam) overnight at 4 °C in a humidified container. After washing in PBS, the sections were incubated with an HRP-conjugated secondary antibody (1:2000, ab97051; Abcam) for 1 hour at room temperature. The sections were further incubated with 2,4-diaminobenzidine substrate and counterstained with hematoxylin.

### Immunofluorescence analysis

After washing in PBS, the sections of each group were blocked in 10% goat serum (16210064; Gibco) for 30 minutes at 37 °C. For double labeling, two compatible primary mouse anti-rabbit antibodies were added: anti-BrdU (1:250, ab8152; Abcam) and anti-α-SMA (1:200, ab7817; Abcam), anti-Collagen I (1:1000, ab90395; Abcam), anti-Collagen III (1:500, ab6310; Abcam), anti-Jagged1 (1:500, ab89663; Abcam), rabbit anti-Notch1 (1:500, ab128076; Abcam), and anti-Hes1 (1:100, ab119776; Abcam). After incubating at 4 °C overnight, the sections were washed with 3% BSA/PBS and incubated with the following secondary antibodies for 1 hour: goat anti-mouse IgG labeled with Alex Fluor 488 (1:200, ab150113; Abcam) and goat anti-mouse IgG labeled with Alexa Fluor 594 (1:200, ab150116; Abcam). Sections were documented with a fluorescence microscope (OLYMPUS, Japan).

### Statistical analysis

Data were analyzed with PRISM5.0 software (GraphPad, CA, USA). Values were expressed as the mean ± standard deviation (SD) unless otherwise indicated. Comparisons of expression difference between control and experimental groups were conducted by Student’s *t* test. The differences between multiple groups were compared using one-way analysis of variance (ANOVA), followed by a Bonferroni post-hoc test for pairwise comparisons. All statistical analyses were performed by SPSS 19.0 software (SPSS, Chicago, IL, USA), and *P* < 0.05 indicates that the difference were statistically significant.

## Results

### bFGF inhibits the differentiation of ESCs to MFB in vitro

After treatment for 10 days, the ESCs of different groups were collected for further experiments. To confirm the effect of bFGF on ESC differentiation to MFB, we tested the expression of α-SMA, CK10, and CK15 by FCM, and detected the expression of α-SMA, Collagen I (Col I), and Collagen III (Col III) by qRT-PCR and western blot analysis. The ratio of CK15 and CK10 reflects the purity of ESCs: the higher the ratio, the higher the purity [23, 24]. α-SMA is a specific marker of MFB and Col I and Col III are metabolites of MFB, and they were used to show the differentiation of ESCs to MFB [25, 26].

**Fig. 1:**
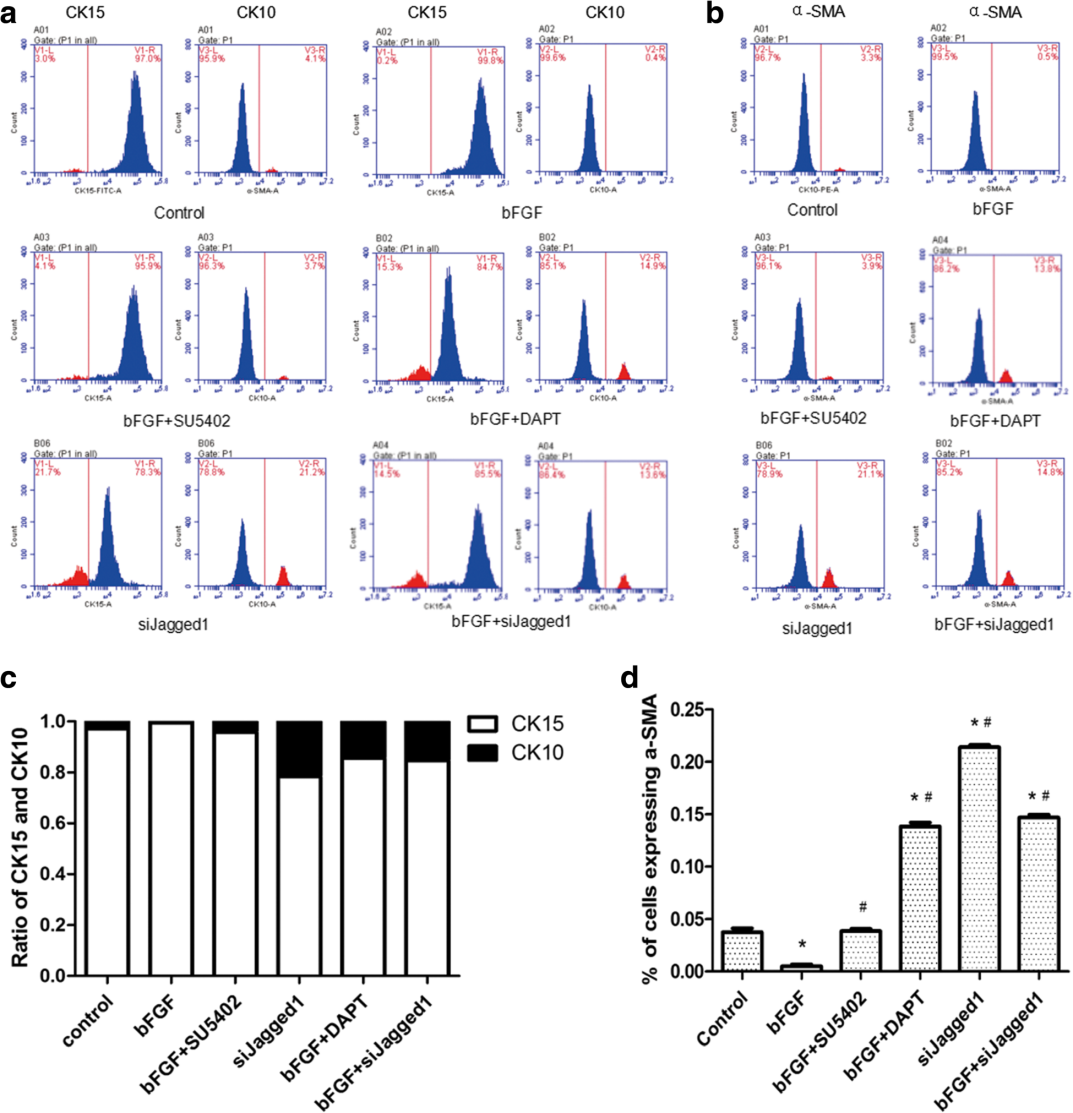
bFGF promoted proliferation of ESCs and inhibited cells’ differentiation to MFB. **a, b** Expression of CK10, CK15, and α-SMA of each group on day 10 detected by flow cytometry analysis. **c, d** Ratio of CK15 and CK10 and expression of α-SMA for each group on day 10 analyzed by Graph Prism 5.0. *Error bars* represent SEM. Student’s *t* test, *P < 0.05 compared with control value (*n* = 5), *#P* < 0.05 compared with bFGF value (*n* = 5)

**Fig. 2:**
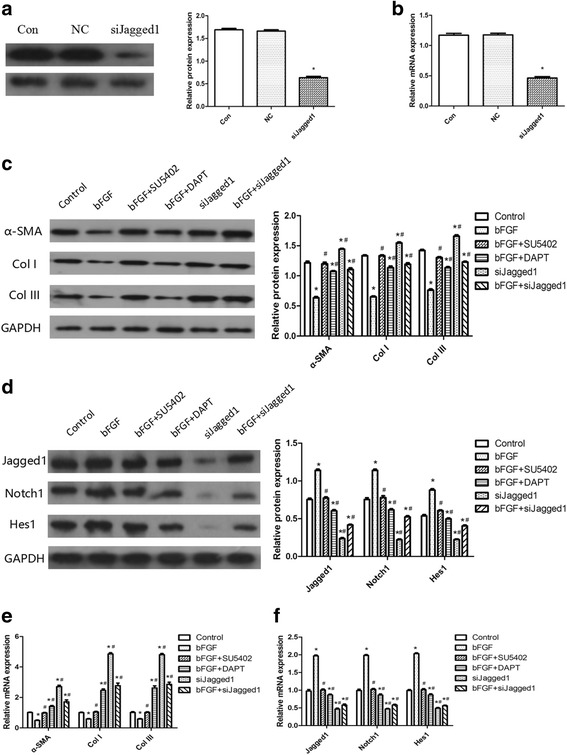
bFGF inhibited differentiation of ESCs to MFB by activating the Notch1/Jagged1 pathway in vitro. **a** Representative immunoblot and results of densitometric analysis of blots showing relative protein levels of Jagged1 in control, negative control (ESCs transfected with a scrambled siRNA for Jagged1), and siJagged1 (ESCs transfected with siRNA specific for Jagged1). GAPDH used as a loading control. **b** Representative qRT-PCR analysis showing relative mRNA levels of Jagged1 in control, negative control, and siJagged1. **c** Representative immunoblot and results of densitometric analysis of blots showing relative protein levels of α-SMA, Col I, and Col III for each group on day 10. **d** Representative immunoblot and results of densitometric analysis of blots showing relative protein levels of Jagged1, Notch1, and Hes1 for each group on day 10. **e** Representative qRT-PCR analysis showing relative mRNA levels of α-SMA, Col I, and Col III for each group on day 10. **f** Representative qRT-PCR analysis showing relative mRNA levels of Jagged1, Notch1, and Hes1 for each group on day 10. **a, b** *P < 0.05. **d–f** *Error bars* represent SEM. Student’s *t* test, *P < 0.05 compared with control value (*n* = 5), *#P* < 0.05 compared with bFGF value (*n* = 5). *Con* control *NC* negative control, *bFGF* basic fibroblast growth factor, *Col* collagen

As FCM results showed, compared with the control group, the ratio of CK15 and CK10 in the bFGF group was significantly higher (*P* < 0.05; Fig. 1a, c), while the expression of α-SMA was obviously lower (*P* < 0.05; Fig. 1b, d). Similarly, in qRT-PCR and western blot detection, the expression of α-SMA, Col I, and Col III was significantly lower in the bFGF group (*P* < 0.05; Fig. 2a, c), while the bFGF + SU5402 group made no obvious difference with the control group (*P* > 0.05; Fig. 2a, c). These results indicated that bFGF could inhibit the differentiation of ESC to MFB in vitro.

### bFGF enhances the expression of Notch1/Jagged1 signaling of ESCs in vitro

To investigate the underlying mechanism of the effect of bFGF on ESCs, the expression of Notch1/Jagged1 signaling-related members was detected by qRT-PCR and western blot analysis. As shown in Fig. 2b, d, compared with the control group, bFGF treatment significantly upregulated the expression of the Notch1 ligand Jagged1 (*P* < 0.05), Notch1 (*P* < 0.05), and Notch1 downstream target Hes1 (*P* < 0.05), while the bFGF + SU5402 group made no obvious difference (*P* > 0.05). These results suggested a possible involvement of the Notch1/Jagged1 pathway in bFGF-induced intracellular changes in ESCs.

### Inhibition of Notch1/Jagged1 pathway suppresses the effects of bFGF on ESCs

To verify the role of the Notch1/Jagged1 pathway in mediating bFGF actions in ESCs, we used DAPT, a specific inhibitor of Notch pathway, to interfere with Notch signaling in ESCs. Furthermore, we also suppressed Jagged1 with specific siRNA siJagged1 to block the Notch1/Jagged1 pathway of ESCs. qRT-PCR and western blot analysis showed that siJagged1 was able to effectively knock down the expression of Jagged1 in ESCs (Fig. 2a, b). As shown in Fig. 2, compared with the control group, expression of α-SMA, Col I, and Col III in the siJagged1 group was significantly higher (*P* < 0.05). Compared with the bFGF group, expression of α-SMA, Col I, and Col III in the bFGF + DAPT group and bFGF + siJagged1 group was significantly higher (*P* < 0.05). These results suggested bFGF might inhibit the differentiation of ESCs to MFB by activating the Notch1/Jagged1 pathway. What is more, we also found that expression of α-SMA, Col I, and Col III in the bFGF + siJagged1 group was higher than in the bFGF + DAPT group (*P* < 0.05), which indicated that Jagged1 ligand might play an important role in the effects of bFGF on ESCs.

### bFGF accelerates wound closure and alleviates scar in vivo

**Fig. 3:**
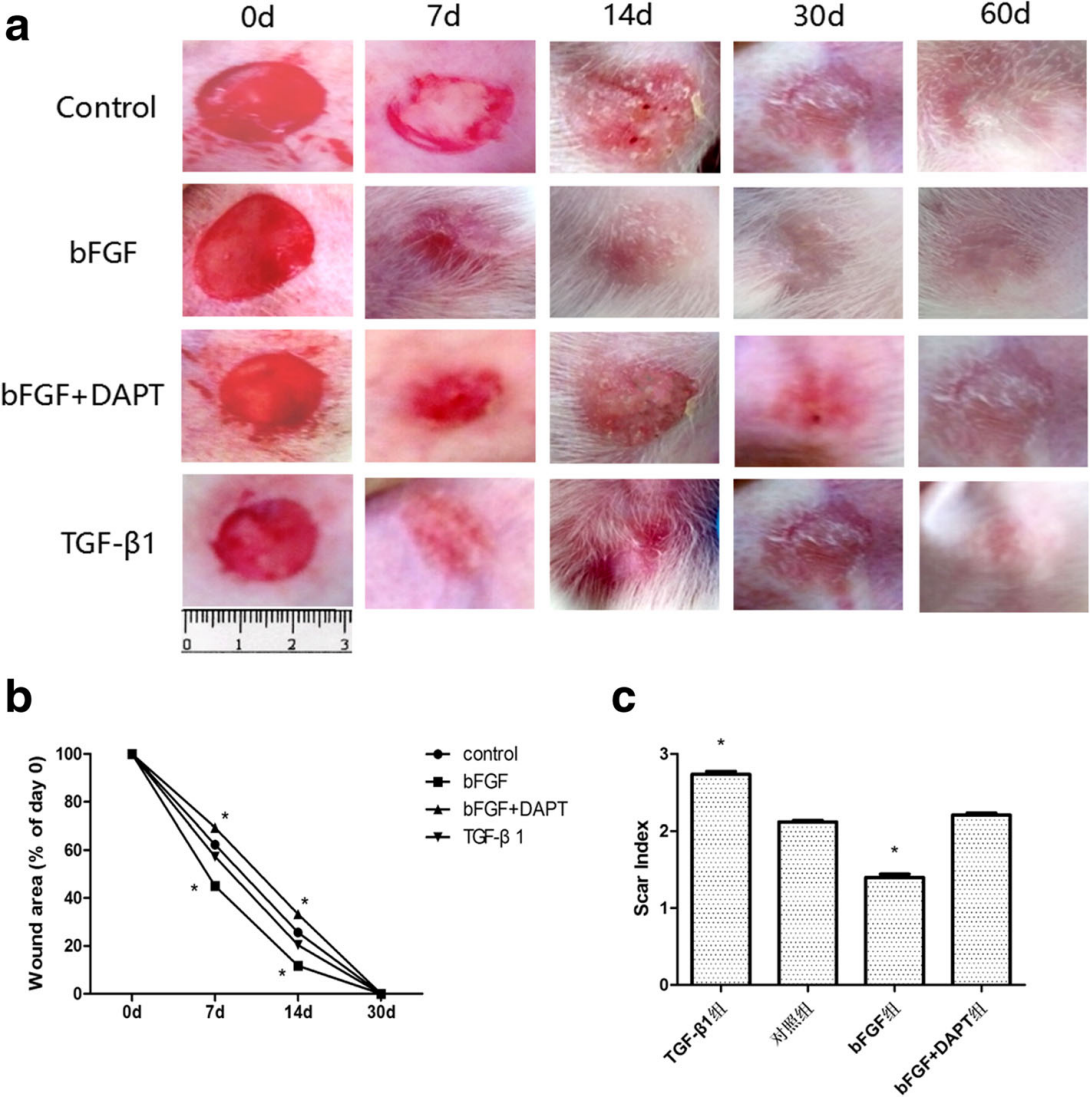
Pharmacological effect of bFGF, TGF-β1, and bFGF + DAPT in repairing wound healing and scaring. Full-thickness dermal wounds were induced in rabbit ears and treated by saline (control), bFGF, bFGF + DAPT, and TGF-β1 respectively. **a** Representative rabbit ear from each group taken on post-injury days 0, 7, 14, 30, and 60. **b** Wound areas for each group. The computation was that the indicated area was divided by the initial area. Results represent means ± SEM. *P < 0.05 compared with control value (*n* = 10). **c** Scar indexes for each group. The computation was that the thickness difference of the scar minus the adjacent normal skin was divided by the adjacent normal skin. Results represent means ± SEM. *P < 0.05 compared with control value (*n* = 10). *d* days, *bFGF* basic fibroblast growth factor

To investigate the role of bFGF in vivo, we conducted our experiments using the rabbit ear scar model. After labeling and random grouping, we treated the wound with bFGF, bFGF + DAPT, TGF-β1, and saline respectively. Meanwhile, we recorded the wound healing time, photographed the wound, measured the wound areas and the thickness of scar tissues, and calculated the residual wound area rate and scar index regularly. As Fig. 3 shows, compared with the control group, the bFGF group showed a significantly shorter healing time (*P* < 0.05; Fig. 3a), lower residual wound area (*P* < 0.05; Fig. 3b), and lower scar index (*P* < 0.05; Fig. 3c), while the bFGF + DAPT group presented an obvious healing delay and higher scar index (*P* < 0.05; Fig. 3). What is more, the GF-β1 group showed a relative shorter wound healing time (*P >* 0.05; Fig. 3a, b) and an obviously higher scar index (*P* < 0.05; Fig. 3c). These results suggested that bFGF could promote wound healing and alleviate scar, while adding DAPT or using TGF-β1 might aggravate scar eventually.

### bFGF promotes re-epithelialization, skin attachment regeneration, and collagen reassignment

**Fig. 4:**
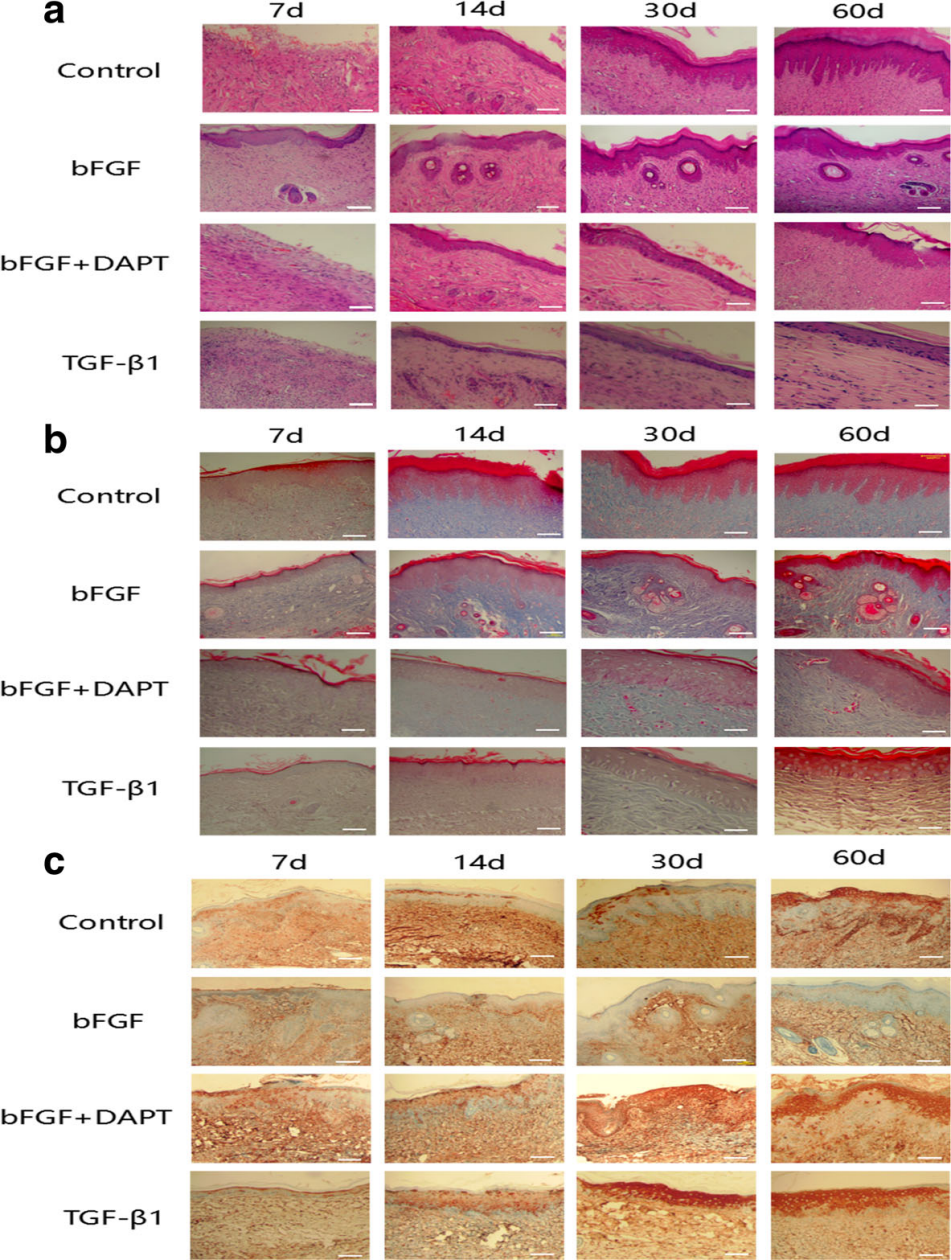
Histological features and expression of α-SMA of the rabbit ear wounds in each group. **a, b** Skin tissue sections stained with H&E and Masson showing histological features in rabbit ears treated with saline (control), bFGF, bFGF + DAPT, and TGF-β1 on post-injury days 7, 14, 30, and 60. bFGF-treated ears exhibited significantly higher quality wound healing, with more cell layers, more epidermal ridges, more formation of primitive hair follicle and sweat gland structures, and more regular and ordered collagen arrangement. Ears treated with bFGF + DAPT and TGF-β1 exhibited poor-quality wound healing compared with control ears. **c** Immunohistochemical staining for α-SMA was performed using skin tissue sections in rabbit ears treated with saline (control), bFGF, bFGF + DAPT, and TGF-β1 on post-injury days 7, 14, 30, and 60. bFGF-treated ears exhibited a significantly higher quality wound healing with a significant decrease of α-SMA, while ears treated with bFGF + DAPT and TGF-β1 exhibited poor-quality wound healing with increase of α-SMA. *Scale bar*, 100 μm. *d* days, *bFGF* basic fibroblast growth factor

To further evaluate the wound healing quality and the scar hyperplasia, we selected the scar tissue specimen at specific time points (7, 14, 30, and 60 days), and observed re-epithelialization, skin appendage regeneration, and collagen reassignment by H&E and Masson staining. As shown in Fig. 4, the wound healing quality of the bFGF group was significantly better than that of the control group, with more cell layers, more epidermal ridges, more formation of primitive hair follicle and sweat gland structures, and more regular and ordered collagen arrangement. However, when the Notch1/Jagged1 pathway was inhibited in the bFGF + DAPT group and TGF-β1 group, the re-epithelialization, skin appendage regeneration, and collagen reassignment were significantly impaired (Fig. 4a, b). These results suggests that bFGF has an active function in promoting re-epithelialization, skin appendage regeneration, and collagen reassignment, and may mainly work through the Notch1/Jagged1 pathway.

### bFGF inhibits differentiation of ESCs to MFB in vivo via the Notch1/Jagged1 pathway

**Fig. 5:**
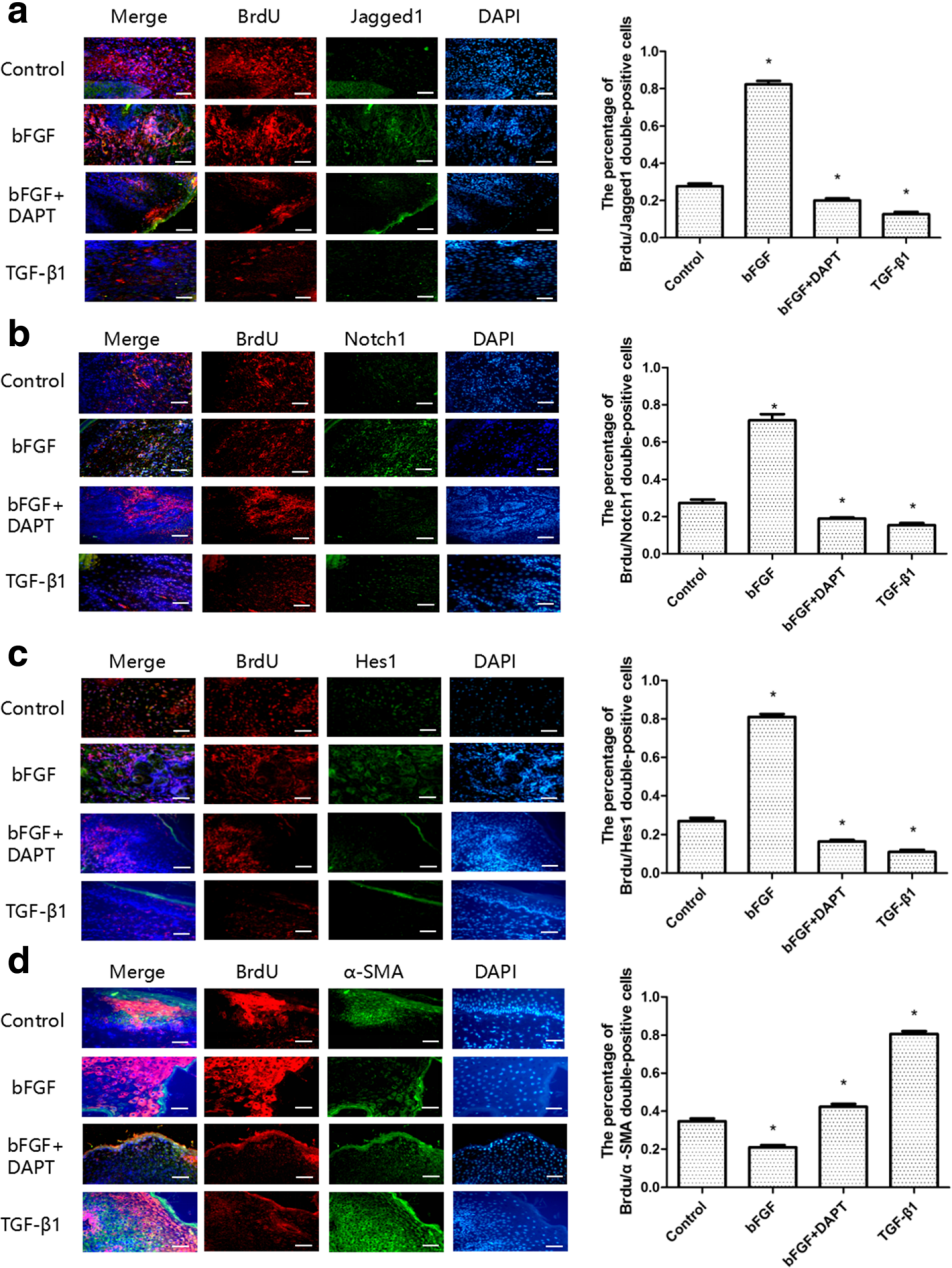
Relationships of bFGF and the Notch1/Jagged1 pathway and differentiation of ESCs in healed skin analyzed by immunofluorescence. **a**, **b, c** Representative Brdu/Jagged1, Brdu/Notch1, and Brdu/Hes1 double-positive cells in healed skin and the percentage of the positive cells to total cells in healed skin of each group on post-injury day 60. **d** Representative Brdu/α-SMA double-positive cells in healed skin and the percentage of the positive cells to total cells in healed skin of each group on post-injury day 60. *Error bars* represent SEM. Student’s *t* test, *P < 0.05 compared with control value (*n* = 10). *Scale bar*, 50 μm. *bFGF* basic fibroblast growth factor

**Fig. 6:**
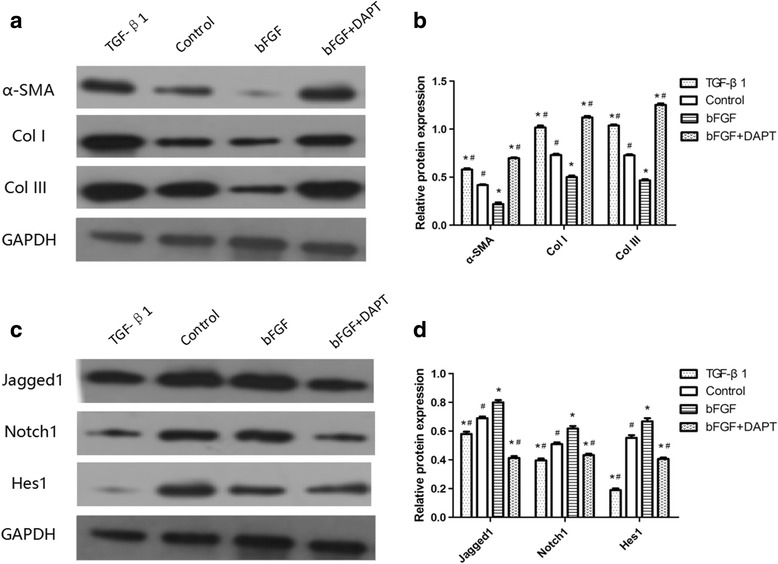
Relationships of bFGF and the Notch1/Jagged1 pathway and differentiation of ESCs in healed skin analyzed by western blot. **a, b** Representative immunoblot and results of densitometric analysis of blots showing relative protein levels of α-SMA, Col I, and Col III for each group on post-injury day 60. **c, d**. Representative immunoblot and results of densitometric analysis of blots showing relative protein levels of Jagged1, Notch1, and Hes1 for each group on post-injury day 60. *Error bars* represent SEM. Student’s *t* test, *P < 0.05 compared with control value (*n* = 10), *#P* < 0.05 compared with bFGF value (*n* = 10). *bFGF* basic fibroblast growth factor, *Col* collagen

By immunohistochemistry staining (Fig. 4c), we found that bFGF can improve the quality of wound healing by inhibiting the expression of α-SMA significantly. To further study its mechanism and verify our hypothesis in vivo, we labeled ESCs with BrdU and detected the relative expression levels of Notch1/Jagged1 signaling components and α-SMA at the same time by double-immunofluorescence staining. As shown in Fig. 5, compared with the control group, the expression of BrdU/Jagged1 (Fig. 5a), BrdU/Notch1 (Fig. 5b), and BrdU/Hes1 (Fig. 5c) double-positive cells in the bFGF group was significantly higher (*P* < 0.05), while expression of BrdU/α-SMA (Fig. 5d) was obviously lower (*P* < 0.05). The double-positive cells were mainly detected in hair follicle cell nucleus and skin basal cell nucleus. On the contrary, expression of BrdU/Jagged1, BrdU/Notch1, and BrdU/Hes1 double-positive cells in the bFGF + DAPT group was significantly lower (*P* < 0.05) than the control group, while expression of BrdU/α-SMA was obviously higher (*P* < 0.05). What is more, to test our results further we examined these indexes by western blot analysis, and consequently found similar results (Fig. 6). These results suggested that bFGF could inhibit ESC differentiation to MFB by activating the Notch1/Jagged1 pathway in vivo.

## Discussion

As a common complication of wound healing, scar has seriously impacted the life of patients for a long time, but there is still no effective treatment [27]. bFGF has been reported to promote wound healing and reduce scarring, but the underlying mechanisms remain unclear [14]. With relevant research going deep, it has been confirmed that the decrease of ESCs and the hyperplasia of MFB are the main causes of scar [28, 29]. In our previous study, we found that bFGF could promote the proliferation and migration of ESCs in vitro. In this study, we demonstrated that bFGF can also inhibit the differentiation of ESCs to MFB by activating the Notch1/Jagged1 pathway. We firstly blocked the Notch1/Jagged1 pathway by adding the relevant inhibitor DAPT and knocking down Jagged1 to confirm our conjecture in vitro, and then verified our results using the rabbit ear scar models. Our results provide the evidence that bFGF reduces scar by inhibiting the differentiation of ESCs to MFB via the Notch1/Jagged1 pathway.

Although the mechanism of scar is still unclear, the hyperplasia of MFB has so far been confirmed to be the most important factor [30]. Early in 2009, Ishiguro et al. [31] identified bFGF as a potent stimulator for the reduction of the myofibroblastic area in vivo, presumably because of its effects on the downregulation of α-SMA expression as well as rapid induction of apoptosis in myofibroblasts. A recently study in cell therapy also found that bFGF could inhibit the differentiation of cardiac stem cells to MFB, which improved extracellular matrix dysregulation post myocardial infarction [32]. While these findings show that that bFGF could reduce the formation of MFB, its effects on ESCs, a key role in wound healing and re-epithelialization, are still unknown [33]. Herein, we speculated that bFGF might inhibit the differentiation of ESCs to MFB analogously. In our in-vitro study, we detected the expression of α-SMA, which is a specific marker of MFB, to show the differentiation of ESCs to MFB directly. At the same time, we also examined the expression of Col I and Col III, which are metabolites of MFB, to reflect the differentiation of ESCs to MFB indirectly. The results from FCM, RT-qPCR, and western blot analysis consistently showed that expression of α-SMA, Col I, and Col III in the bFGF group was significantly lower than in other groups, suggesting that bFGF could inhibit ESC differentiation to MFB.

The Notch1/Jagged1 pathway has been confirmed to play an important role in stem cells [34]. As shown in research conducted by Guiu et al. [35], Hes1 repressors are essential regulators of hematopoietic stem cell development downstream of Notch signaling. By activating its downstream gene *Hes1*, the Notch1/Jagged1 pathway mainly promotes stem cell proliferation and inhibits their differentiation [36]. In our previous study, we found that activating the Notch1/Jagged1 pathway contributed to promoting ESC proliferation and inhibiting the cells’ differentiation [11], which is consistent with the effects of bFGF on ESCs. Therefore we supposed that bFGF might regulate ESCs by activating the Notch1/Jagged1 pathway. In our present in-vitro study, we first detected the expression of Jagged1, Notch1, and Hes1 for each group, and found this was much higher in the bFGF groups than in other groups. We then adopted two ways to interfere with the Notch1/Jagged1 pathway: adding DAPT and knocking down Jagged1. As a γ-secretase inhibitor, DAPT can inhibit the Notch pathway by blocking the cleavage of NICD, which is necessary for activation of transcription of downstream target genes [20]. To further detect the function of the Notch1/Jagged1 pathway on ESCs, we knocked down Jagged1 ligands by transfecting the corresponding siRNA into ESCs [37]. By inhibiting the Notch1/Jagged1 pathway, we observed that the effects of bFGF on ESCs were significantly weakened. What is more, this effect decrease was much more obvious in the bFGF + siJagged1 group than in the bFGF + DAPT group, suggesting that bFGF might work mainly by activating Jagged1. However, in our present study we found that the siJagged1 group exhibited a significant difference from the bFGF + DAPT group and bFGF + siJagged1 group, which indicates that there is another signaling pathway existing between bFGF and the differentiation of ESCs. This result is consistent with our previous findings that both the Wnt and Notch pathways are important to wound healing [22].

Since Morris et al. [21] found that rabbits’ ears could be used for studying scar, the rabbit ear wound model has been an ideal model to date [38]. In order to verify our findings in vivo, we conducted our animal research on the rabbit ear wound models. By observing the wound healing time and measuring the wound areas and the thickness of scar tissues, we found that bFGF could obviously promote wound healing and reduce the scar. These results are consistent with the function of bFGF in clinical observation. As we all know, normal skin tissue is constituted by the epidermis, dermis, and skin appendages [39]. At the junction of epidermal and dermal tissue, we often see a clear papillary structure and basal cells, in which the ESCs reside [40]. In our in-vivo study, we collected the tissues of different groups in 60 days for pathology detection, and found that bFGF significantly promoted re-epithelialization, skin attachment regeneration, and collagen reassignment, with clearer cell layers, more epidermal ridges, and more reformation of hair follicle and sweat gland structures. Recent studies have shown that the Notch1/Jagged1 pathway is involved in regulating investigation of hair follicles into the dermis and maintaining postnatal hair homeostasis [41] and that DAPT could modulate human hair follicle stem cell proliferation and differentiation [42], and we found that the Notch1/Jagged1 pathway also played an important role in the process of bFGF on wound healing. When we inhibited the Notch signal pathway by DAPT, the function of bFGF on wound healing was weakened significantly. This result was analogous to the study of Chen et al. [43], which confirmed that Notch1 signaling inhibits apoptosis of human dental follicle stem cells via both the cytoplasmic mitochondrial pathway and nuclear transcription regulation.

In order to study the effect of bFGF on ESCs in vivo, we labeled ESCs by injecting BrdU. Because ESCs possess a longer period between divisions, they should be the only cells retaining the BrdU label after a long chase period [22]. The specific mechanism for retaining the label is that stem cells normally remain in a relatively static state of long-term, slow differentiation, in which BrdU dilutes much more slowly than in other cells. Therefore, after labeling for a certain period of time, only slowly proliferating cells such as ESCs retain the BrdU marker [44]. In our present study, we collected the scar tissue specimens of different groups in 60 days and detected the double-positive cells of BrdU/Jagged1, BrdU/Notch1, BrdU/Hes1, and BrdU/α-SMA simultaneously by double-immunofluorescence staining. In line with our expectation, we found that bFGF could obviously activate the Notch1/Jagged1 pathway, while inhibiting the differentiation of ESCs to MFB at the same time. Moreover, we further detected these indices by western blot analysis and observed the same results.

Additionally, we discovered that TGF-β1 played an absolutely opposite role with bFGF during wound healing and scar formation in rabbit ears. Although the TGF-β1/Smad3 pathway has been widely accepted to be a major factor leading to scar [45], our study showed that the Notch1/Jagged1 pathway was obviously suppressed in the TGF-β1 group, which led to an overdeposition of MFB and eventually aggravated the scar. In recent research, Luo [46] stated that signaling crosstalk exists between TGF-β1/Smad and other signaling pathways, which could properly account for our results. This discovery not only indicates that TGF-β1 might increase the scar by inhibiting the Notch1/Jagged1 pathway, but also suggests that there is a close relationship between various cytokines and signaling pathways during wound healing and scar hyperplasia, which remains to be studied further.

## Conclusion

In summary, this work provides the first evidence that bFGF can reduce scar by promoting the proliferation of ESCs and inhibiting its differentiation to MFB by activating the Notch1/Jagged1 pathway. Moreover, we demonstrate that TGF-β1 might aggravate the scar by inhibiting the Notch1/Jagged1 pathway. This work not only builds a bridge between the signaling pathway and ESCs in wound healing, but also reveals the formation of scar in a new view, which may help us explore new approaches to prevent and treat scar. However, our work just begins. The relation of the Notch1/Jagged1 pathway with other cytokines such as TGF-β1 and its crosstalk with different pathways remain to be studied further.

## Abbreviations

bFGF: Basic fibroblast growth factor
Brdu: 5-Bromodeoxyuridine
DAPT: *N*-[*N*-(3,5-Difluorophenacetyl)-l-alanyl]-*S*-phenylglycine *t*-butyl ester
ESC: Epidermal stem cell
FCM: Flow cytometry
FITC: Fluorescein isothiocyanate
GAPDH: Glyceraldehyde-3-phosphate dehydrogenase
H&E: Hematoxylin and eosin
Hes: Hairy and enhancer of split
HRP: Horseradish peroxidase
K-SFM: Keratinocyte serum-free medium
NICD: Notch intracellular domain
qRT-PCR: Quantitative real-time polymerase chain reaction
SD rats: Sprague–Dawley rats
TGF-β1: Transforming growth factor-β1
WB: Western blot
α-SMA: α-Smooth muscle actin
